# Robotic versus laparoscopic hysterectomy; comparison of early surgical outcomes

**DOI:** 10.4274/jtgga.galenos.2020.2019.0187

**Published:** 2020-12-04

**Authors:** Özgüç Takmaz, Mete Güngör

**Affiliations:** 1 Department of Obstetrics and Gynecology, Acıbadem Mehmet Ali Aydınlar University Faculty of Medicine, İstanbul, Turkey

**Keywords:** Robotic hysterectomy, laparoscopic hysterectomy

## Abstract

**Objective:**

To compare early surgical outcomes of robotic assisted laparoscopic hysterectomy with laparoscopic hysterectomy for benign diseases, in terms of operation time, estimated blood loss (EBL), perioperative complications, hospital stay and first gas discharge.

**Material and Methods:**

Medical records of 146 patients who either underwent laparoscopic (n=84) or robotic assisted laparoscopic hysterectomy (n=62) for benign diseases were extracted from records. Demographic characteristics and operation time, EBL, length of hospital stay and first gas discharge were compared between the groups.

**Results:**

Mean age and mean body mass index of both groups were comparable. The difference in the mean EBL was not statistically significant between laparoscopic (91±65 mL) and robotic group (80±37 mL, p=0.43). The difference in the mean first gas discharge time was not statistically different between laparoscopic (15±5 hours) and robotic group (17±6 hours, p=0.33). The length of hospital stay was comparable between groups (1.4±0.5 vs 1.5±0.7 days, p=0.64). The mean operation time was longer for the robotic group (150±180 minimum) when compared with laparoscopic group (105±18 minimum, p<0.01). The mean uterine weight of the robotic group was significantly heavier compared with laparoscopic group (234±157 grams vs 153±119 grams, respectıvely, p<0.01).

**Conclusion:**

Early surgical outcomes of robotic assisted laparoscopic and laparoscopic hysterectomy were comparable in terms of EBL, first gas discharge and hospital stay. Operation time was longer for robotic hysterectomy.

## Introduction

Hysterectomy is still the second most common gynecologic procedure for benign uterine diseases second to c-section (1). The most common indications for hysterectomy are fibroids and abnormal uterine bleeding (2). Various novel types of medical and surgical treatments have been increasingly implemented in gynecology practice including for hysterectomy. Hysterectomy may be performed with abdominal (AH), vaginal (VH), laparoscopic (LH) and robotic assisted laparoscopic (RH) approaches. An increasing trend for minimally invasive hysterectomy approaches using the latter three techniques, VH, LH and RH, has occurred in the last two decades (3). Compared to AH, minimally invasive hysterectomy procedures provide shorter hospital stay, less bleeding, faster recovery and lower infection rates with better cosmetic results (4,5). As a result, minimally invasive hysterectomy procedures are recommended as the first option when compared with the abdominal route (6). After the Food and Drug Administration (FDA) approval of robotic assisted laparoscopic surgery in gynecologic procedures in 2005, another alternative option was accepted into the range of minimally invasive hysterectomy procedures available. Although RH has disadvantages, such as increased cost and longer operation times, improved dexterity, faster learning curve, instrument facilitation of 7 degrees of freedom, decreased tremor and 3D visualization make RH procedure preferable, especially in more difficult cases such as in morbidly obese patients, having had prior abdominal surgery or patients with an enlarged uterus (7,8,9).

In this study retrospective comparison of the perioperative outcomes of patients undergoing either LH or RH patients who had undergone hysterectomy for benign gynecologic indications was investigated.

## Material and Methods

Medical records of the patients who underwent RH or LH between January 2015 and June 2018 for benign indications were extracted from the hospital database system. Benign indications consisted of fibroids, chronic pelvic pain, abnormal bleeding or uterine prolapse. The study was approved by institutional review board ethics committee (ATADEK 2019-12). Patients who had a non-gynecologic or gynecologic additional procedure in the same session or who had a history of prior surgery or with chronic non-gynecologic conditions (liver, kidney, pulmonary disease, diabetes) were excluded from the study groups. All procedures performed in the study were in accordance with the ethical standards of the institutional and/or national research committee and with the 1964 Helsinki declaration and its later amendments or comparable ethical standards. For undergoing surgery written informed consent was obtained from all participants.

Medical records of operation time, estimated blood loss (EBL), length of hospital stay and first gas discharge time were evaluated and compared between the groups. Operation time was defined as the time from intubation to the end of extubation of the patient. EBL was calculated as the difference in fluid volume between irrigation and suction. Hospital stay was defined as the post-operative days passed after surgery until discharge. First gas discharge time was defined as in which hour the first gas discharge was recorded after the surgery. Uterine weight was recorded by weighing the excised uterus in the pathologic examination room immediately after removal.

A Rumi II (Cooper Surgical, Trumbull, CT, USA) uterine manipulator was used in all cases after intubation. All operations were performed in the lithotomy position with steep Trendelenburg (up to 30 degrees) with 13mmHg carbon dioxide pressure.

LH operations were performed via four abdominal ports (10 mm umbilical, 5 mm right, left and suprapubic port), and integrated advanced bipolar and ultrasonic instrument (Thunderbeat-Olympus Corp. of America 3500 Corporate Parkway, Center Valley, PA 18034, U.S.A.) was used for dissecting and vessel sealing.

RH operations were performed with either a da Vinci Si^R^ or da Vinci Xi^R^ (Intuitive Surgical, Inc., Sunnyvale, CA., USA) platform via four abdominal ports which were: for the Si platform - 10 mm umbilical, 8 mm right and left ancillary ports and 12 mm assistant port; and for the Xi platform - 8 mm umbilical, right and left ancillary ports and 12 mm assistant port). Side docking was performed for applying the patient card to abdominal ports in order to manage the uterine manipulator. Monopolar scissors were used for dissection and bipolar fenestrated forceps were used for vessel sealing.

After prophylactic antibiotic administration, all cases underwent the same surgical steps. Following the port placement, firstly the round ligaments were dissected. Then the infundibulopelvic ligaments were dissected and if the patient was under 50 years old, utero-ovarian ligaments were dissected in order to preserve the ovaries. Bilateral uterine arteries were sealed and dissected after skeletonization. After incising the vaginal cuff, hysterectomy tissues were removed through the vagina. Vaginal cuff closures were performed with a 2.0 barbed suture in both groups.

No major complication was recorded during any operation or in the early post-operative periods. After post anesthesia care unit, all patients were followed up in the gynecology inpatient service with administration of a routine post-operative follow up medication consisting of non-steroid analgesics and anti-emetics.

### Statistical analysis

The R-3.4.3 programme (R-Core Team. 2017, The R Foundation, https://www.r-project.org/) was used for statistical analysis. Normality assessment was made using the Shapiro-Wilks test. Descriptive statistical methods (mean, standard deviation, median) were used for evaluating the study data. Student’s t-test was used to compare normally distributed quantitative variables, while Mann-Whitney U test was used for non-normally distributed variables. The statistical significance level was set at 0.05.

## Results

Medical data of 146 patients were extracted for the study groups. Of the 146 patients, 84 (57.5%) underwent LH and 62 (42.5%) underwent RH.

Table 1 shows the demographic and surgical characteristics of the two groups. Mean age and body mass index (BMI) were not significantly different between groups. Operation time was significantly longer in the RH group compared to the LH group (150 min ± 180 vs 105 min ± 18, respectively, p<0.01). Uterine weight was significantly higher in RH group than LH group (234±157 vs 153±119 grams, respectively, p<0.01). The mean EBL were 80 mL and 91 mL for the RH and LH groups, respectively, which was not significantly different (p=0.43). The mean first gas discharge time after the operation in the RH group was 17 hours, while in the LH group it was 15 hours and, again, this was not significantly different (p=0.33). The mean hospital stay durations were not statistically different between the RH group and LH group (1.5±0.7 and 1.4±0.5 days, respectively, p=0.64).

## Discussion

In the present study perioperative outcomes for RH were comparable with LH, in terms of bleeding, first gas discharge time and hospital stay in patients who underwent simple hysterectomy for benign conditions. However, operation time was significantly longer in the RH group than the LH group. In addition, uterine weight was significantly greater in the RH group compared to the LH group.

After the first description of total laparoscopic hysterectomy by Reich et al. (10) in 1989, the application of minimally invasive procedures increased in hysterectomy operations. Various studies revealed the advantages of minimally invasive hysterectomy, such as less bleeding, lower peri-operative and post-operative complication rates, shorter hospital stay and shorter post-operative recovery period (11,12,13). Not only were peri-operative improvements evident, long-term benefits of minimally invasive hysterectomy procedures were also reported (5). Despite the advantages of minimally invasive hysterectomy procedures, some drawbacks, such as a steeper learning curve, increased need for a greater range of equipment and more education for hospital staff in the new techniques, have slowed the acceptance of these procedures into routine practice.

One of the most important improvements in minimally invasive gynecologic surgery was the introducing of robotic surgery. The first reported cases series of RH was published in 2002 (14). Thanks to the endo-wrist movements and three dimensional visualization, robotic surgery is superior to laparoscopic procedures in terms of precise dissection and accurate suturing. A further advantage of RH is the shorter learning curve. Studies have shown that as few as fifty RH procedures are sufficient experience to complete the learning curve for this technique (15,16). In addition, following FDA approval for RH, the widespread acceptance of this technique accelerated (17). However, robotic surgery has some disadvantages. These are longer operation times and higher costs (18,19,20). Longer operation times are due to the docking procedure, that is the fixation of the robotic arms to the ports. It has been shown that docking times can be reduced with greater experience (21). Increased cost is the other major disadvantage of robotic surgery. The average cost of RH is 1.5-3 times higher than the average cost of the LH (22). Investment in the console, maintenance costs and instrument costs per case are the main three contributors to the increased cost of robotic procedures. However, increase in the frequency of usage and decrease in equipment production costs may reduce the average cost of RH in the long term.

Another disadvantage of RH is the size of the robotic system components. A robotic surgery system has three components; the surgeon console, the patient card and the endoscopic tower. In order to organize and apply these devices effectively, both a large operating room and trained hospital staff are needed. There are also cosmetic disadvantages when using robotic surgery. In robotic gynecological surgery, the upper abdominal or umbilical area has to be used for port placements. Port incisions are also larger than laparoscopic incisions. Goebel and Goldberg (23) suggested that robotic surgery may be less preferable because of the poorer cosmetic outcomes associated with its use.

Although discomfort of the surgeon is not a component of perioperative outcome, it is another disadvantage of robotic surgery. Neck stiffness, and finger and eye fatigue have been reported as common complaints of robotic surgeons (24). However, there is no trial that has compared surgeon discomfort between RH and LH operations.

Hospital stay is another component of the perioperative outcome. Similarly; to previous reports, in our study hospital stay for LH and RH was comparable (25).

Although, no perioperative complication was reported in our study groups, a meta-analysis reported that vaginal cuff dehiscence may be higher in RH (26). However, Scandola et al. (27) reported that RH was associated with lower perioperative complications in terms of vaginal cuff dehiscence. When considering peri-operative and post-operative complications, the vaginal approach may be considered as an alternative minimally invasive technique. A Cochrane analysis of hysterectomy techniques highlighted the fewest intra-operative complications, quickest return to baseline activity, and the fewest number of urinary/bowel dysfunction and dyspareunia issues with the vaginal approach (28).

Despite these disadvantages, there are studies showing that robotic hysterectomy is preferable in some patient groups. Several studies have reported that the use of robotic surgery is more advantageous than laparoscopy, especially in obese patients or those having a large uterus (7,29,30,31).

### Study Limitation

There are some limitations of our study. As our study did not include an AH group, the perioperative improvements of endoscopic methods which were reported in previous studies could not be confirmed. Another limitation is the difference of the uterine weight between the groups. Greater uterine weight may have been a cause of the longer operation times in the RH group in our study but, as reported, RH may be preferable in patients with a larger uterus (7,29,30,31).

## Conclusion

RH did not improve perioperative outcomes in patients who underwent simple hysterectomy for benign conditions in this cohort. As operation times were longer and RH is associated with significantly increased costs, it does not seem reasonable to choose a robotic approach for simple hysterectomy. Our results confirm the American College of Obstetricians and Gynecologists guidelines which recommend vaginal or laparoscopic hysterectomy for simple hysterectomy (32). However, robotic hysterectomy is an important minimally invasive surgical alternative for laparoscopic hysterectomy, depending on the patient's status, especially with regard to patient BMI, the difficulty of the surgery and the preferences of the surgeon.

## Figures and Tables

**Table 1:**
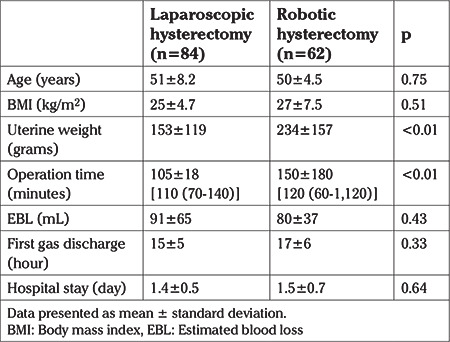
Early surgical parameters and characteristics of groups

## References

[1] Whiteman MK, Hillis SD, Jamieson DJ, Morrow B, Podgornik MN, Brett KM, et al (2008). Inpatient hysterectomy surveillance in the United States, 2000-2004. Am J Obstet Gynecol.

[2] Merrill RM (2008). Hysterectomy surveillance in the United States, 1997 through 2005. Med Sci Monit.

[3] Turner LC, Shepherd JP, Wang L, Bunker CH, Lowder JL (2013). Hysterectomy surgery trends: a more accurate depiction of the last decade?. Am J Obstet Gynecol.

[4] Olsson JH, Ellstrom M, Hahlin M (1996). A randomised prospective trial comparing laparoscopic and abdominal hysterectomy. Br J Obstet Gynaecol.

[5] Nieboer TE, Hendriks JC, Bongers MY, Vierhout ME, Kluivers KB (2012). Quality of life after laparoscopic and abdominal hysterectomy: a randomized controlled trial. Obstet Gynecol.

[6] Johnson N, Barlow D, Lethaby A, Tavender E, Curr E, Garry R (2006). Surgical approach to hysterectomy for benign gynaecological disease. Cochrane Database Syst Rev.

[7] Orady M, Hrynewych A, Nawfal AK, Wegienka G (2012). Comparison of robotic-assisted hysterectomy to other minimally invasive approaches. JSLS.

[8] Alkatout I, Mettler L, Maass N, Ackermann J (2016). Robotic surgery in gynecology. J Turk Ger Gynecol Assoc.

[9] Varghese A, Doglioli M, Fader AN (2019). Updates and controversies of robotic-assisted surgery in gynecologic surgery. Clin Obstet Gynecol.

[10] Reich H, DeCaprio J, McGlynn F (1989). Laparoscopic hysterectomy. J Gynecol Surg.

[11] Garry R, Fountain J, Mason S, Hawe J, Napp V, Abbott J, et al (2004). The evaluate study: two parallel randomised trials, one comparing laparoscopic with abdominal hysterectomy, the other comparing laparoscopic with vaginal hysterectomy. BMJ.

[12] Jacoby VL, Autry M, Jacobson G, Domush R, Nakagawa S, Jacoby A (2009). Nationwide use of laparoscopic hysterectomy compared with abdominal and vaginal approaches. Obstet Gynecol.

[13] Gobern JM, Rosemeyer CJ, Barter JF, Steren AJ (2013). Comparison of robotic, laparoscopic, and abdominal myomectomy in a community hospital. JSLS.

[14] Diaz-Arrastia C, Jurnalov C, Gomez G, Townsend C Jr (2002). Laparoscopic hysterectomy using a computer-enhanced surgical robot. Surg Endosc.

[15] Sandadi S, Gadzinski JA, Lee S, Chi DS, Sonoda Y, Jewell EL, et al (2014). Fellowship learning curve associated with completing a robotic assisted total laparoscopic hysterectomy. Gynecol Oncol.

[16] Seamon LG, Fowler JM, Richardson DL, Carlson MJ, Valmadre S, Phillips GS, et al (2009). A detailed analysis of the learning curve: robotic hysterectomy and pelvic-aortic lymphadenectomy for endometrial cancer. Gynecol Oncol.

[17] Pasic RP, Rizzo JA, Fang H, Ross S, Moore M, Gunnarsson C (2010). Comparing robot-assisted with conventional laparoscopic hysterectomy: impact on cost and clinical outcomes. J Minim Invasive Gynecol.

[18] Paraiso MF, Ridgeway B, Park AJ, Jelovsek JE, Barber MD, Falcone T, et al (2013). A randomized trial comparing conventional and robotically assisted total laparoscopic hysterectomy. Am J Obstet Gynecol.

[19] Sarlos D, Kots L, Stevanovic N, von Felten S, Schar G (2012). Robotic compared with conventional laparoscopic hysterectomy: a randomized controlled trial. Obstet Gynecol.

[20] Lonnerfors C, Reynisson P, Persson J (2015). A randomized trial comparing vaginal and laparoscopic hysterectomy vs robot-assisted hysterectomy. J Minim Invasive Gynecol.

[21] Martinez-Maestre MA, Gambadauro P, Gonzalez-Cejudo C, Torrejon R (2014). Total laparoscopic hysterectomy with and without robotic assistance: a prospective controlled study. Surg Innov.

[22] Tapper AM, Hannola M, Zeitlin R, Isojarvi J, Sintonen H, Ikonen TS (2014). A systematic review and cost analysis of robot-assisted hysterectomy in malignant and benign conditions. Eur J Obstet Gynecol Reprod Biol.

[23] Goebel K, Goldberg JM (2014). Women's preference of cosmetic results after gynecologic surgery. J Minim Invasive Gynecol.

[24] Lee GI, Lee MR, Green I, Allaf M, Marohn MR (2017). Surgeons' physical discomfort and symptoms during robotic surgery: a comprehensive ergonomic survey study. Surg Endosc.

[25] Albright BB, Witte T, Tofte AN, Chou J, Black JD, Desai VB, et al (2016). Robotic versus laparoscopic hysterectomy for benign disease: a systematic review and meta-analysis of randomized trials. J Minim Invasive Gynecol.

[26] Uccella S, Ghezzi F, Mariani A, Cromi A, Bogani G, Serati M, et al (2011). Vaginal cuff closure after minimally invasive hysterectomy: our experience and systematic review of the literature. Am J Obstet Gynecol.

[27] Scandola M, Grespan L, Vicentini M, Fiorini P (2011). Robot-assisted laparoscopic hysterectomy vs traditional laparoscopic hysterectomy: five meta-analyses. J Minim Invasive Gynecol.

[28] Aarts JW, Nieboer TE, Johnson N, Tavender E, Garry R, Mol BW, et al (2015). Surgical approach to hysterectomy for benign gynaecological disease. Cochrane Database Syst Rev.

[29] Nawfal AK, Orady M, Eisenstein D, Wegienka G (2011). Effect of body mass index on robotic-assisted total laparoscopic hysterectomy. J Minim Invasive Gynecol.

[30] Orady ME, Karim Nawfal A, Wegienka G (2011). Does size matter? The effect of uterine weight on robot-assisted total laparoscopic hysterectomy outcomes. J Robot Surg.

[31] Payne TN, Dauterive FR, Pitter MC, Giep HN, Giep BN, Grogg TW, et al (2010). Robotically assisted hysterectomy in patients with large uteri: outcomes in five community practices. Obstet Gynecol.

[32] No authors listed (2015). Committee opinion no. 628: robotic surgery in gynecology. Obstet Gynecol.

